# Breast metastasis from lung adenocarcinoma: a case report and review of the literature

**DOI:** 10.3389/fonc.2024.1370453

**Published:** 2024-05-22

**Authors:** Jialing Ding, Huayan Gu, Zhi Yang, Yiqiao Lu, Guilong Guo

**Affiliations:** Department of Breast Surgery, The First Affiliated Hospital of Wenzhou Medical University, Wenzhou, Zhejiang, China

**Keywords:** lung cancer, breast metastasis, breast cancer, diagnosis, differentiation

## Abstract

Lung cancer (LC) is one of the most lethal and most prevalent malignant tumors, and lung adenocarcinoma (LUAD) is the most common pathological type of lung cancer. Breast cancer (BC) is the most common cancer worldwide, but metastases to the breast from extramammary neoplasms are rare, especially from the lung. Early diagnosis and differentiation of primary from metastatic breast carcinoma are essential. Here, we present a case of metastases to the breast from lung adenocarcinoma, the treatment options varied according to disease progression.

## Introduction

Female breast cancer has overtaken lung cancer as the most common diagnosed cancer worldwide (1). According to the statistics of the American Cancer Society (2), lung cancer has occupied the first place of cancer deaths (21% in both sexes), and breast cancer is second only to lung cancer in women. However, metastases to the breast from extramammary neoplasms are not very common (3). Koch et al. conducted a statistical analysis of 463 cases and obtained a proportion of breast metastases incidence from primary cancers rate: melanoma (29.8%), bronchial lung cancer (16.4%), gynecological cancer (12.7%), gastrointestinal cancer (9.9%), leukemia and lymphoma (8.4%), sarcoma (7.3%), and renal cancer (1.5%). Differentiating between primary breast cancer and breast metastases is a challenge, particularly when the metastases are identified as lung adenocarcinoma. At present, the main means include clinical manifestation, imaging characteristics, histological examination, and immunohistochemistry testing (IHC). The choice of combination treatment for primary and metastatic breast cancer is also different depending on the primary focus and pathology. Here, we present a case of metastases to the breast from lung adenocarcinoma and its treatment.

## Case report

**Figure 1:**
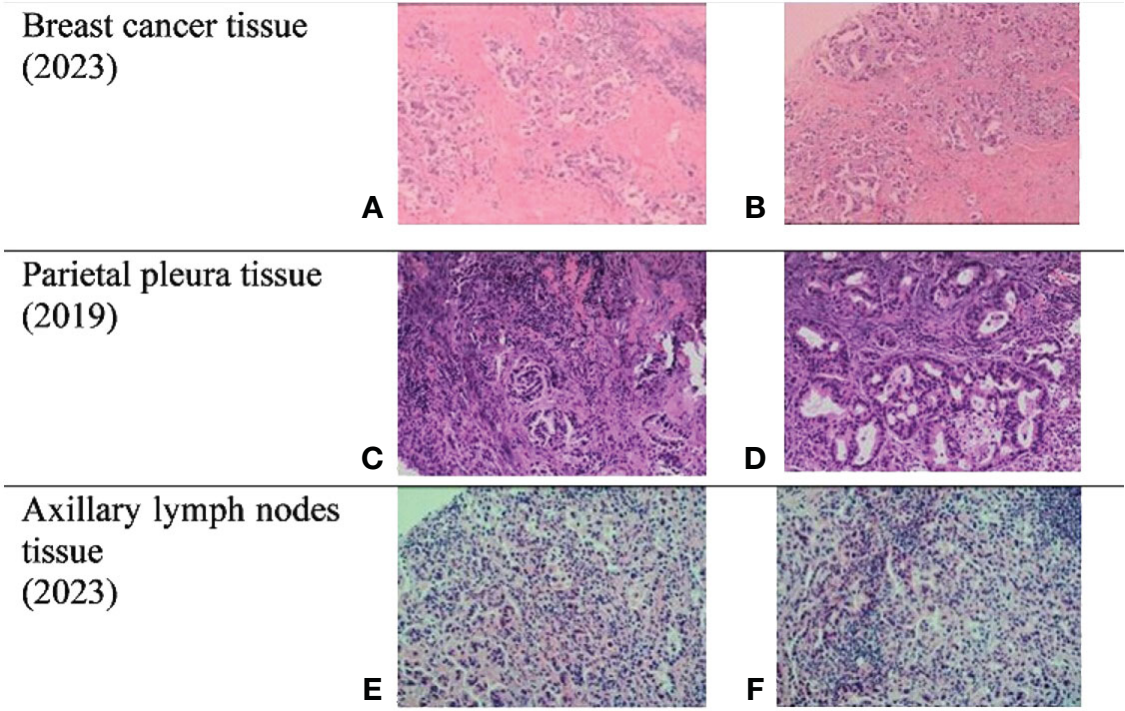
Biopsies. **(A, B)** Breast cancer tissue from mammotome biopsy. **(C, D)** Parietal pleura tissue from pleuroscopy biopsy. **(E, F)** Axillary lymph nodes (ALN) tissue from core needle biopsy. ER and PR were both negative in these three tissues, and NapsinA and TTF-1 were both positive in them.

In February 2019, a 52-year-old woman with no history of smoking attended to our hospital complaining of recurring chest distress. Computed tomography (CT) scan revealed an abnormal mass and pleural effusion in her left lung. Subsequently, she underwent pleuroscopy and biopsy. Based on epidermal growth factor receptor (EGFR) mutational analysis, the specimen retrieved at pleuroscopy was identified as pulmonary adenocarcinoma with a mutation in exon-19 19-Del of the EGFR gene. Then, she received icotinib (125 mg orally three times daily) for therapy. However, 1 year later, the patient developed recurring plural effusion that was confirmed to be malignant plural effusion with T790 positivity, which indicated that she had developed a resistance mutation. Therefore, she changed to receive Osimertinib (AZD-9291) for therapy. In July 2022, as the disease progressed, she began to accept pemetrexed plus carboplatin and bevacizumab for 10 cycles of chemotherapy. Unfortunately, 4 years after the diagnosis of lung cancer, the patient’s physical examination revealed breast lesions. Previously, the patient had no breast skin changes and no palpable breast lumps or local pain. The ultrasonic examination detected nodules in the left breast with calcifications (BI-RADS 4 degree), accompanied with bilateral axillary lymph node enlargement and left supraclavicular lymph node enlargement. She underwent minimally invasive mammotome biopsy (**Figure 1**), and the biopsy specimen revealed estrogen receptor (ER)-negative, progesterone receptor (PR)-negative, and HER2-negative invasive ductal carcinoma. Considering the patient’s history of lung cancer, we conducted additional immunohistochemical tests on her and found that TTF-1 and Napsin-A results were positive, which indicated that the lung cancer had metastasized to the breast, so we chose to use albumin-bound paclitaxel plus cisplatin and Sintilimab for therapy. In the subsequent telephone follow-up, the patient was actively receiving lung cancer treatment, but we did not obtain effective breast examination information. The patient had no prior or family history of breast cancer before.

## Discussion

Early diagnosis and differentiation of primary and metastatic breast cancer are important for timely treatment. Clinical history, imaging, and pathological findings should be taken into account in differentiating secondary mass from primary breast cancer. Previous studies have shown that the most common features of breast metastases present as palpable, rapidly growing, well-circumscribed, and painless breast masses with predilection to the upper outer quadrant. Unlike primary breast cancer, most metastatic breast cancers, while located on the surface, do not manifest as contractions of the skin or nipples (4). Imaging studies differential diagnosis relies on X-ray, ultrasound, and CT. For the wide range of imaging manifestations of the metastatic lesions, it may be extremely difficult to distinguish them if only based on mammographic findings. The absence of micro-calcifications is considered a characteristic of metastatic lesions to the breast, with the exception of ovarian cancer (5). There are very few studies in the literature on MRI findings for breast metastases.

If the diagnosis is based solely on clinical manifestations and imaging, it is easy to make mistakes. Immunohistochemistry plays a crucial role in diagnosis by using specific markers to distinguish primary breast cancer. According to the World Health Organization (WHO) classification, breast cancer (BC) is classified into many subtypes, as defined by IHC testing of ER, PR, HER2, and Ki67 status (6). Based on both molecular and histological evidence, BC could be categorized into three groups: BC expressing hormone receptor [estrogen receptor (ER+) or progesterone receptor (PR+)], BC expressing human epidermal receptor 2 (HER2+), and triple-negative breast cancer (TNBC) (ER−, PR−, and HER2−). The treatment approaches should be based in the BC molecular characteristics (7). ER, PR, and Her-2 were all negative in metastatic breast cancer, which was easily confused with TNBC. Therefore, for patients with a history of malignant tumors, relevant markers of the primary lesion should be added to the immunohistochemical test. Specific immunohistochemical markers for lung carcinoma include thyroid transcription factor-1 (TTF-1), Napsin-A, and CK7. Since no single marker is complete sensitive and false negative results are inevitable, we must use a panel of markers to improve accuracy. The double stain of TTF-1 and Napsin A was proposed for increased sensitivity and specificity for lung cancer. We conducted a comprehensive analysis of the existing literature, including 29 reported cases with detailed immunohistochemical results and our case. (**Table 1**) TTF-1 was positive in 26 breast biopsies. Only two breast biopsies were negative for TTF-1 and Napsin A, and their lung biopsy markers were also negative, which was consistent. In Takayo Ota’ report (25), the patient initially had no detectable immunohistochemical markers specific to lung cancer and was therefore misdiagnosed with a double cancer. However, researchers analyzed her tissues again after her death and again found the same EGFR mutation, which she corrected for metastatic lung adenocarcinoma. In our report, the patient’s IHC testing revealed ER−, PR−, and HER2− invasive ductal carcinoma, similar to TNBC. However, TTF-1, NapsinA, and CK7 indexes were all positive, indicating metastatic breast cancer rather than primary TNBC. Combined with her clinical history, she was finally diagnosed with lung adenocarcinoma and breast metastasis. It is important to note that not everyone with a history of malignant tumors can be diagnosed with metastatic tumors, which can be both cancers at the same time. For example, a woman with a 1-year history of non-small-cell lung cancer came to our hospital with a painless breast mass. Combined with hematoxylin–eosin and immunohistochemical results, the final diagnosis was primary invasive breast cancer with positive ER and PR expression, and radical mastectomy and axillary dissection were performed. The case of metastatic breast cancer described in this report does not require surgery. In addition to surgery therapy, there are also principled differences in drug selection between primary breast cancer and metastatic breast tumor with extramammary cancer. Primary breast cancer is undoubtedly targeted at breast lesions; the treatment of metastatic breast disease is mainly focused on the primary lesion, so the treatment plan for patients with different primary lesions is often different. Targeted therapy and immunotherapy are currently emerging strategies that can target metastatic tumors, but research is still incomplete and their contribution to reducing mortality is not significant (26). It can be concluded that distinguishing metastases breast cancer from primary breast carcinoma is essential because of therapeutic and prognostic significance. Multiple aspects must be combined in diagnosis, especially characteristic immune markers.

**Table 1:** Breast lesion metastasis from primary lung cancer: literature review 2000–2023.

	Age	Sex	IHC markers of breast biopsy	IHC markers of lung biopsy
**Gómez-Caro A(2006) (** 8)	65	Male	CK4− CK7− TTF-1−	CK4− CK7− TTF-1−
**Rimner A(2007) (** 9)	81	Female	TTF-1+ ER− PR− HER-2−	TTF-1+
**Fulciniti F(2007) (** 10)	59	Female	TTF-1+ ER− PR−	NA
**Klingen TA(2009) (** 11)	79	Female	TTF-1+ CK7+	NA
	70	Male	TTF-1+ CK7+	NA
	54	Female	TTF-1+ CK7+	NA
**Maounis N(2010) (** 12)	73	Female	TTF-1+ SP-A+ GCDFP15− ER− mammaglobin−	TTF-1+ CK 5/6− ER− CA-125-thyroglobulin−
**Fukumoto K(2011) (** 13)	65	Female	TTF-1+ ER− EGFR-19 (E746-A750) mutation	EGFR-19 (E746-A750) +
**Ko K (2012) (** 14)	47	Female	TTF-1+ ER− PR− mammaglobin−	NA
**Sato K (2012) (** 15)	57	Female	TTF1+ CK7+ SP-A+ CK20− GCDFP15− ER− PR− HER-2−	NA
**Ji FF (2012) (** 16)	40	Female	TTF-1+ ER− PR− HER2− GCDFP15− mammaglobin−	NA
	49	Female	TTF-1+ ER− PR− HER2− GCDFP15− mammaglobin−	NA
**Sousaris N(2013) (** 17)	55	Female	TTF-1+ napsin A+ ER− PR− HER-2−	NA
**Huang HC(2013) (** 18)	70	Female	TTF-1+ GCDFP15− ER− PR−	EGFR-21-L858R+
**Liam CK (2013) (** 19)	70	Female	TTF-1+ ALK− ER− PR− HER-2−	TTF-1+ ALK− Exon-20 T790M+
**Hachisuka A(2014) (** 20)	60	Male	TTF-1− Napsin-A− SP-A− ER− PR− GCDFP15−	TTF-1- Napsin-A− SP-A− ER− PR− GCDFP15−
**Dansin E,(2015) (** 21)	52	Female	TTF1+ GATA3− GCDFP15− PAX8− ER− PR-HER2−	Exon-19 19-Del +
**Shen YW (2015) (** 4)	52	Female	TTF-1+ Napsin A+ CK7+ ER− PR− GCDFP15−	TTF-1+ CK7+ Napsin A+ CK20− CK5/6− GCDFP15−
**Lin Q (2016) (** 22)	51	Male	TTF-1+ Chomogranin A+ Synaptophysin+ CD56+ ER− GCDFP15− HER−	TTF-1+ Synaptophysin+ CD56+ EGFR-21-L858R+
**Ninan J (2016) (** 23)	67	Female	TTF-1+ Napsin A+ CK7+ GATA-3− GCDFP15−	NA
**Cserni G (2017) (** 24)	60	Female	TTF-1+ Napsin A+ CK7+ GATA-3− PR− HER2− mammaglobin−	TTF1+ CK7+ p63−
**Ota T (2018) (** 25)	69	Female	ER− PR− HER2− EGFR-21-L858R+	EGFR-21-L858R+
**Enrico D (2019) (** 5)	29	Female	TTF-1+ CK7+ Napsin A+ AE1AE3+ P63− CK20− ER− PR− GATA3− HER2−	TTF-1+ CK7+ AE1AE3+ Napsin A+ P63− CK20−
**our (2023)**	52	Female	TTF-1+ Napsin A+ CK7+ ER− PR− HER2− Mammaglobin− GATA3− GCDFP15− CK20−	TTF-1+ Napsin-A+ CK7+ CD56− CgA− Syn−

NA, not available (not mentioned in patient’s report).

Accurate diagnosis and treatment are inseparable. In clinical practice, it is necessary to closely monitor the progress of advanced malignant tumors and make accurate diagnoses in a timely manner to avoid incorrect treatment. In the future, cancer treatment will move towards an era of precision, comprehensiveness, and individualization.

## Data availability statement

The raw data supporting the conclusions of this article will be made available by the authors, without undue reservation.

## Ethics statement

Written informed consent was obtained from the individual(s) for the publication of any potentially identifiable images or data included in this article.

## Author contributions

JD: Conceptualization, Data curation, Formal analysis, Funding acquisition, Investigation, Methodology, Project administration, Resources, Software, Supervision, Validation, Visualization, Writing – original draft, Writing – review & editing. HG: Data curation, Formal analysis, Funding acquisition, Investigation, Methodology, Supervision, Writing – review & editing. ZY: Data curation, Formal analysis, Funding acquisition, Supervision, Validation, Writing – review & editing. YL: Data curation, Investigation, Resources, Software, Validation, Visualization, Writing – review & editing. GG: Conceptualization, Investigation, Methodology, Project administration, Resources, Software, Supervision, Validation, Visualization, Writing – review & editing.
